# Staff empowerment and engagement in a magnet® recognized and joint commission international accredited academic centre in Belgium: a cross-sectional survey

**DOI:** 10.1186/s12913-018-3562-3

**Published:** 2018-10-03

**Authors:** Peter Van Bogaert, Danny Van heusden, Stijn Slootmans, Ingrid Roosen, Paul Van Aken, Guy H. Hans, Erik Franck

**Affiliations:** 1 Nursing and Midwifery Sciences, Centre for Research and Innovation in Care (CRIC), Faculty of Medicine and Health Sciences University of Antwerp Belgium, Universiteitsplein 1, B-2610 Wilrijk, Antwerpen Belgium; 2 Nursing Department, Antwerp University Hospital, Wilrijkstraat 10, B-2650 Edegem, Antwerpen Belgium; 3 Department of Quality and Patient Safety, Antwerp University Hospital, Wilrijkstraat 10, B-2650 Edegem, Antwerpen Belgium; 4 Department of Algology and Evidence Based Medicine, Multidisciplinary Pain Centre, Antwerp University Hospital, Wilrijkstraat 10, B-2650 Edegem, Antwerpen Belgium; 5 Department of healthcare, Karel De Grote University College Antwerp Belgium, Brusselstraat, 45 2018 Antwerpen, Belgium

**Keywords:** Empowerment, Engagement, Burnout, Magnet recognition, Accreditation, Quality and patient safety

## Abstract

**Background:**

A substantial number of studies linked aspects of a balanced, healthy and supportive nurse practice environment with quality and patient safety. To what extent balanced work characteristics such as social capital, decision latitude and workload are relevant for all staff engaged in patient care including healthcare and medical staff in a Magnet Recognized and Joint Commission International accredited academic centre is unclear. The study aim is to investigate associations between work characteristics such as social capital, decision latitude and workload, work engagement and feelings of burnout as explanatory variables and job satisfaction, turnover intentions and perceived quality of care as dependent variables in a study population of nursing, healthcare and medical staff taken in account generation differences.

**Methods:**

Hierarchical regression analysis estimated strength of associations with demographic characteristics (block-1), professional category (block-2), work characteristics (block-3) and work engagement or burnout dimensions (block-4) as explanatory variables of job satisfaction and turnover intention and quality of care as outcome variables.

**Results:**

The study confirmed and extended previous study findings demonstrating positive impact on staff’ job outcomes and assessed quality of care by *balanced work characteristics* such as social capital, decision latitude and workload in nursing staff (*N* = 864), healthcare staff (*N* = 131) and medical staff (*N* = 241). Generational characteristics and professional category were associated with turnover intentions and less favorable assessed quality of care, respectively. Explained variances of studied models ranged from 14.4 to 45.7%.

**Conclusion:**

Engaging and committing staff to promote excellent patient outcomes in daily interdisciplinary practice works through clear frameworks, methods and resources supported by governance and policy structure that makes outcomes visible and accountable.

## Background

During the last decade significant changes and transformations in healthcare are ongoing. Medical practices in hospitals are gradually evolved in more interdisciplinary collaborations between various healthcare professions and hospital services creating complex processes with multiple stakeholders and ownership such as in the care of patients with cancer [1]. Transformations because of technology and biomedical sciences as well as changes in patient populations with more chronic conditions based on non-communicable diseases make healthcare services challenging [2]. Meanwhile costs and quality become more important in healthcare, both as an accountable process for governments and patients [3]. Hospitals, dealing with the introduction of new treatments, technologies and complex processes, are in constant change model [4]. Currently 4 generations (rather than 2 or 3 typical in previous eras) make up the workforce. Observation suggests that each generation holds different values and that some of these values may be in conflict as well among these differences are alternative views on work–life balance that affects a person’s sense of how much work is reasonable [5]. Therefore, hospitals need the governance and policy as well as the staff that can adapt necessary and inevitable changes and transformations accurately focusing on patient and families’ needs [6, 7]. The effort and work that hospitals perform internationally to improve patient care processes and patient outcomes through an accountable and visible process such as accreditation and certification by external bodies is remarkable [8, 9]. Moreover, the American Nurses Credentialing Centre Magnet Recognition Program® has established international attention and recognition for nursing excellence and improved outcomes [10, 11]. A program initial developed to attract and retain nurse workforce with a strong focus on a sustainable culture that promotes and establish tangible improved patient outcomes along accurate structure and process outcomes.

Little information is available on effective accreditation strategies as well as evidence that support the effect on patient outcomes or other important markers such as core measures, organizational culture nor reliability [12]. A nationwide study of a census of public hospitals in Denmark identified improvements in the quality of hospital care where the base line hospital performance was below best practice target values following the introduction of an accreditation program [13]. In addition, the decrease in trend post- accreditation was noted and authors suggested that hospitals’ focus on improvement was affected by the external pressure which follows the on-site survey. A European study conducted in 89 hospitals in 6 countries reveals that quality and safety structures and procedures were more evident in hospitals with either the type of external assessment and identified more impact on hospital management, patient safety and clinical practice in accredited hospital then ISO-certified hospitals [9]. Authors notified study limitations such as the sample size and confounded variations in the application and certification within and between countries. A systematic review of hospital accreditation reveals a lack of studies that report intervention context, implementation, or cost as well as how accreditation is managed and executed, and the varied financial and organizational healthcare constraints. The strategies hospitals should implement to improve patient safety and organizational outcomes related to accreditation and certification components remains also unclear [12].

Our research program focus on organizational features of nurses’ workplaces in relation to nurse and patient outcomes aiming to provide evidence for organizational context of nursing practices that support and adapt changes proactively in practices within inevitable hospital and healthcare transformations. Our study findings identified *balanced work characteristics* comparable with the empowerment concept such as social capital, decision latitude and workload as essential in nurse work environments [14]. Organizational empowerment is a construct based on Kanters’ model of structural empowerment that described workers’ access to relevant information, support, and resources needed to do the job as well as opportunities to learn and grow as necessary resources [15]. Moreover, research confirmed the insight that the extent of job demands and the presence of resources reflect in either strain processes through feelings of burnout with negative impact on wellbeing and productivity as loss cycles, or motivational processes through engagement and involvement with positive impact on wellbeing and productivity as gain cycles [16–18]. A substantial number of studies identified and linked aspects of a balanced, healthy and supportive nurse practice environments [19, 20] with quality and patient safety indicators [21–23]. In our previous studies unfavorable rated social capital expressed by a lack of support of peers, shared values and mutual trust; unfavorable rated decision latitude because of limited abilities to make decisions and limited capacity to use and develop professional and personal skills, along with high workloads are strongly associated with low morale and engagement with a negative impact on professional wellbeing and assessed outcomes [24, 25]. To what extent balanced work characteristics such as social capital, decision latitude and workload are relevant for all staff engaged in patient care including healthcare and medical staff in a Magnet Recognized and Joint Commission International accredited academic centre is unclear. In 2007, the study hospital’ Chief Executive Officer (CEO) and Chief Nursing Officer (CNO) along with the hospital board have decided to unroll major changes gradually in the hospital policy and governance [26]. This study describes a component of our research program, in which we guided and evaluated hospital and nursing unit interventions to enhance nursing performance and quality patient care. Therefore, the study’s aim is to investigate associations between work characteristics such as social capital, decision latitude and workload, work engagement and feelings of burnout as explanatory variables and job satisfaction, turnover intentions and perceived quality of care as dependent variables in a study population of nursing staff, healthcare staff and medical staff taken in account demographics such as gender and generation differences.

## Methods

A cross-sectional study was performed in a 600-bed academic acute care centre in the Dutch-speaking part of Belgium. Nursing staff, healthcare staff and medical staff (*N* = 2359) were invited to fill in voluntarily an online provided questionnaire during a period of 8 weeks (March 15th and April 28th 2017).

### Study context

In a first step (2007) a long-term transformation process of the hospital organizational form from *hierarchical and departmental* to one that was *flat*, where team-decisions prevail, and *interdisciplinary*, with mutual respect amongst all disciplines, supported by *participative and visible management style* was set up [26]. This phase was underpinned and inspired by research evidence on professional nurse retention and attraction [27, 28] and the principles of the ANCC Magnet Recognition Program® [29, 30] to create practice environments conducive to professionalism, retention, productivity, safe and high-quality patient care. In a second step (2011), the Productive Ward – Releasing Time to Care™ program or PW program was introduced as an integral part of a hospital-wide governance policy to provide structural supports for nursing care and quality improvement processes [31]. The UK National Health Service (NHS) Institute has developed the PW program for Innovation and Improvement, a program that was launched in 2007 [32]. Meanwhile the hospital provided structural support for data-driven improvement projects through project management approach and Plan-Do-Study-Act based projects [7] for all clinical as well as technical units such outpatient clinic, OR and ER. In the same period, nurse-sensitive patient outcomes were quarterly reported to the US National Database of Nursing Quality Indicators [33] to benchmark outcome indicators such as central line associated blood stream infection (CLABSI), catheter associated urinary tract infections (CAUTI), hospitals acquired pressure injuries (HAPI) and falls with injury. The benchmarking of these four patient outcome indictors was part of the designation process to ANCC Magnet Recognition® the hospital aspired as a journey to nursing excellence and improved outcomes [34]. In a fourth step (2012) the hospital became involved in an accreditation process, the Joint Commission International or JCI, as a part of a larger governmental hospital accountability strategy [35]. In Flanders almost all hospitals (except for a dozen) are involved in an accreditation trajectory such as JCI or Qmentum [36, 37]. The accreditation process started with a gap-analysis to evaluate in what extend standards was met in practices followed by a mock survey (2014). August 15th 2015 and October 23th 2017 the hospital received JCI accreditation and was Magnet Recognized® respectively, the latter to our knowledge being the only hospital across Europe.

### Study population

Study population was a convenient sample of staff engaged in patient care such as nursing staff (*N* = 864 or 65% response rate) including registered nurses *N* = 668 (84.3%), midwifes *N* = 49 (5.7%), licensed practice nurses *N* = 83 (9.6%) and nurse managers *N* = 64 (7.4%); healthcare staff (*N* = 131 or 62% response rate) including pharmacists *N* = 9 (6.9%), audiologists and speech therapists *N* = 18 (13.7%), dieticians *N* = 13 (9.9%), psychologists *N* = 20 (15.3%), physiotherapists and occupational therapists *N* = 18 (13.7%), social workers *N* = 11 (9.2%), various therapists and technicians *N* = 42 (32.1%); and medical staff (*N* = 241 or 30% response rate) including medical specialist trainees *N* = 36 (14.5%), independent staff *N* = 39 (16.2%), regular and senior regular staff *N* = 91 (37.8)% and medical department chairs and co-chairs *N* = 75 (31.1%). Although a relatively small group with a limited education level in comparison with the other professional groups, licensed practice nurses were included in the study populations because of their strong integration in care processes at team level and involvement in quality improvement projects.

### Variables and instruments

Survey measurements were selected and validated in previous research projects [25, 14]. The survey measurements were carefully developed and published during the last 10 years with various study populations (primarily nursing in various domains such as acute care, psychiatric care and residential aged care) and various steps: 1) translation procedure [38], factor analysis (explorative and confirmatory) and associations tested within two models (burnout and engagement) [24, 39]; 2) multilevel analyses at team level [40, 41]; 3) confirmation of the tested models by qualitative studies [42, 43] and 4) longitudinal studies evaluating improvement initiative such as productive ward program [26, 31]. To meet potentially biased responding [44] the survey data was a part of management data in the study hospital, although independent conducted as well as all respondents were thoroughly informed and aware of the study results as a part of improvement projects such as productive ward program structurally underpinned and supported by JCI-accreditation the Magnet® recognition program.

*Work characteristics* [39] were measured using three measurement scales tapping *social capital* (6 items), the extent of shared values and perceived mutual trust within teams and organizations [45]; *decision latitude* (6 items), the ability to make decisions, be creative, and use and develop their professional and personal skills at the workplace; and assessed *workload* (7 items) [20, 46]. Respondents rated their agreement or disagreement on 4-point Likert-type scales (*strongly disagree, disagree, agree, strongly agree)*. *Work Engagement* was investigated with the shortened 9-item version Utrecht Work Engagement Scale (*UWES)* [47, 48] and yields 3 separate dimensions’ vigor, dedication, and absorption: *Vigor* is defined as high levels of energy and mental resilience at work. *Dedication* is described as strong involvement in one’s work accompanied by feelings of enthusiasm and significance. *Absorption* relates to being fully engrossed in one’s work and having difficulties detaching oneself from it. The *Maslach Burnout Inventory* [49, 50] is a three-subscale measure including emotional exhaustion (eight items), reflecting one’s depletion of emotional resources and diminution of energy; depersonalization (five items), reflecting one’s negative attitudes and feelings as well as insensitivity and lack of compassion towards patients; and personal accomplishment (seven items), reflecting one’s evaluation of their work related to their feelings of competence. On both tools, respondents rated the frequency of various job-related feelings on 7-point Likert-type scales ranging from *never* to *every day*.

Respondents were asked to rate their perceived *quality of care* overall at *the unit*, and *in the hospital* over the last year on a 4-point Likert-type scales (*poor, fair, good, excellent*). Finally, three types of *job outcomes* were assessed: satisfaction with the current job (*very dissatisfied, dissatisfied, satisfied, and very satisfied*), intention to leave the hospital within the next year (*yes, no*), and intention to leave the nursing profession (*yes, no*) as originally used by Aiken and colleagues [51] and validated in our studies [50]. Cronbach alpha’s value showed internal consistency and reliability of studied variables in nursing staff, healthcare staff and medical staff ranged from 0.71 to 0.92, except for decision latitude (0.63) and depersonalization (0.50) in healthcare staff and depersonalization (0.66) in nursing staff.

All variables, with the exception of workload, emotional exhaustion and depersonalization were coded for analysis with higher scores indicating stronger agreement or more favorable ratings. Cutoffs for high to very high mean scores for each burnout and work engagement dimension were determined by norms studied in Dutch study populations [49].

### Ethical considerations

A qualified ethics review committee, Antwerp University Hospital – University of Antwerp Belgium, approved the study on November 14th 2016 (reference number 14/42/428).

### Data analysis

Demographic characteristics were examined such as gender and generational differences in baby boomers born ≤1964; X between 1965 and ≤ 1979; Y between 1980 and ≤ 1992 [52] and Z ≥ 1993 [53]. To identify relevant associations on respondents who rated outcome variables as favorable as well as strongly favorable we have chosen to treat the outcome variables job satisfaction and quality of care binary. Firstly, respondents who were satisfied or very satisfied versus dissatisfied or very dissatisfied with their job; rated the quality of care at the unit good or excellent and in the hospital improved or strongly improved versus at the unit fair or poor and in the hospital deteriorated or strongly deteriorated. Secondly, respondents who are very satisfied versus satisfied or dissatisfied or very dissatisfied with their job; rated the quality of care at the unit excellent and in hospital strongly improved versus at the unit good or fair or poor and in the hospital improved or deteriorated or strongly deteriorated. The second analyses identified relevant associations of the respondents who rated outcome variables strongly favorable or a population of ambassadors necessary to support and meet high standards of care and quality improvement continuously. Hierarchical regression analysis, based on previous model testing that described associations between predicting, mediating and outcome variables [14, 39] and identified variables for each block, estimated the strength of the associations with demographic characteristics (baby boomers and female as indicators) (block-1), professional category (medical staff as indicator) (block-2), work characteristics (block-3) and work engagement or burnout dimensions (block-4) as explanatory variables of job satisfaction and turnover intention and quality of care as outcome variables (see Tables 2, 3, 4 and 5).

We did not treat missing data and missed cases were not involved in the calculation of each dimension scores (work characteristics, burnout and engagement dimensions). The sample sizes involved in the regression analyses (see Tables 2, 3, 4 and 5) ranged from 1085 (12,5% missing cases) to 1021 (17,3% missing cases), largely comparable between professional categories. A statistical significance level of *P* < .05 was set and the Statistical Package for the Social Sciences (SPSS Inc., Chicago; IBM SPSS statistics Armonk, NY) version 24.0 software was used for all the analyses.

## Results

Table 1 summarizes demographics and study variables.

**Table 1:** Demographics and study variables

	Nursing staff *n* = 864		Healthcare staff *n* = 131		Medical staff *n* = 241	
	n	%	n	%	n	%
Generation Z	72	8.4	3	2.3	0	0.0
Generation Y	275	32.1	64	49.2	82	34.6
Generation X	290	33.8	42	32.3	99	41.8
Babyboomers	221	25.8	21	16.2	56	23.6
Gender (Female)	713	82.5	106	80.9	114	47.3
Satisfied - very satisfied	761	88.1	120	93.0	216	89.6
Very satisfied	226	26.2	43	32.8	74	30.7
Intention to leave hospital	49	5.7	9	6.9	28	11.6
Intention to leave occupation	78	9.0	13	9.9	6	2.5
Quality of care unit good excellent	728	84.3	104	79.4	213	88.4
Quality of care unit excellent	194	22.5	23	17.6	110	45.6
Quality hospital improved certainly improved	596	69.0	102	77.9	210	87.1
Quality hospital centainly improved	75	8.7	9	6.9	28	11.6
	mean	SD	mean	SD	mean	SD
Social capital	3.06	0.54	2.98	0.57	3.05	0.59
Decision latitude	3.10	0.35	3.12	0.35	3.20	0.37
Workload	2.97	0.53	2.75	0.44	2.91	0.52
Vigor	4.53	1.29	4.51	1.01	4.56	1.14
Dedicaton	5.02	1.10	4.96	0.98	5.03	1.05
Absorption	4.44	1.36	4.26	1.30	4.45	1.22
Emotional exhaustion	1.71	1.20	.83	.83	5.10	.79
Depersonalisation	1.70	1.06	.60	.55	5.07	.80
Personal accomplishment	1.98	1.26	1.13	.97	5.11	.74

Social capital, decision latitude, workload range 1–4; work engagement and burnout range 0–6

Two out of three respondents represented generation X (*N* = 586) and generation Y (*N* = 620). One out of four represented baby boomers and nearly 6% represented generation Z (*N* = 96). The latter generation was represented only in nursing staff and healthcare staff. In healthcare staff generation Y represented almost 50% and in medical staff generation X represented almost 42%. In nursing staff and healthcare staff > 80% were female while nearly 50% in medical staff.

The study population satisfaction including very satisfied with the current job ranged from 88.1 to 93% and very satisfied ranged from 26.2 to 32.8%. Intention to leave the hospital and the profession ranged from 5.7 to 11.6% and 2.5 to 9.9%, respectively. Quality of care at the unit (good or excellent) and in the hospital over the last year (improved or certainly improved) ranged from 79.4 to 88.4% and 69.0% to 87.1%, respectively. While excellent and certainly improved assessments ranged from 17.6 and 45.6% and 6.9 to 11.6%, respectively.

Decision latitude and social capital were rated predominately favorable (> 3.0), while workload was rated rather unfavorable (> 2.90) in nursing staff and medical staff and rather moderate (2.75) in healthcare staff. One out of four in nursing staff (*N* = 211) and healthcare staff (*N* = 30) and one out of three in medical staff (*N* = 79) rated emotional exhaustion high and very high. Depersonalization was rated 15.2% (*N* = 131) in nursing staff, 4.6% (*N* = 6) in healthcare staff and 23.3% (*N* = 56) in medical staff as high and very high. However, personal accomplishment was rated by > 70% of the respondents as high and very high (nursing staff *N* = 425, healthcare staff *N* = 94 and medical staff *N* = 174). Moreover, two out of three medical specialist trainees rated emotional exhaustion (*N* = 23) and depersonalization (*N* = 24) as high and very high and almost 70% (*N* = 25) rated high very high personal accomplishment scores.

Half of the respondents rated high and very high on vigor (nursing staff *N* = 480, healthcare staff *N* = 66 and medical staff *N* = 125). Between 65 and 69% rated high and very high on dedication (nursing staff *N* = 599, healthcare staff *N* = 86 and medical staff *N* = 161). More than 60% rated high and very high on absorption (nursing staff *N* = 525, healthcare staff *N* = 75 and medical staff *N* = 147).

In the work engagement and burnout models (see Tables 2, 3 and 4) generation Y and X (block – 1) were significant associated with intention to leave the profession with odds of > 4 and > 9 respectively. The professional categories nursing staff and healthcare staff (block – 2) were associated with intention to leave the profession with odds of > 3 and > 7, respectively. Moreover, nursing staff and healthcare staff (block – 2) had significant less favorable assessed quality of care variables (odds ranged from 50 to 81%).

**Table 2:** Hierarchical regression analyses with personal characteristics (1), category (2), social capital, decision latitude and workload (3) and work engagement dimensions (4) (explanatory variables) and job satisfaction; intention to leave hospital and profession (dependent variables)

Job satisfaction: satisfied or very satisfied (1) versus dissatisfied or very dissatisfied (0)							Job satisfaction: very satisfied (1) versus satisfied or dissatisfied or very dissatisfied (0)						
	B	SE	OR	95% C.I.		adjR^2^		B	SE	OR	95% C.I.		adjR^2^
*N* = 1081				Lower	Upper		*N* = 1081				Lower	Upper	
Generations							Generations						
Generations (Z)	.035	.597	1.04	.32	3.33		Generations (Z)	.182	.356	1.20	.60	2.41	
Generations (Y)	−.546	.310	.58	.32	1.06		Generations (Y)	−.097	.231	.91	.58	1.43	
Generations (X)	−.196	−.322	.82	.44	1.54		Generations (X)	−.155	.225	.86	.55	1.33	
Gender male)	−.012	.278	.99	.57	1.70	.020	Gender (male)	.218	.209	1.24	.83	1.88	.008
Professional Category							Professional Category						
Category (1)	−.612	.300	.54	.28	1.04		Category (1)	−.049	.233	.95	.60	1.50	
Category 2)	.079	.501	1.08	.41	2.89	.035	Category 2)	.453	.319	1.57	.84	2.94	.012
Social capital	.770	.214	2.16***	1.42	3.29		Social capital	.918	.175	2.51***	1.78	3.53	
Decision latitude	.489	.364	1.63	.80	3.33		Decision latitude	1.817	.281	6.15***	3.55	10.67	
Workload	−.608	.233	.54**	.35	0.86	.185	Workload	−1.07	.183	.34***	.240	.49	.275
Vigor	.055	.121	1.06	.83	1.34		Vigor	.130	.136	1.14	.870	1.49	
Dedication	.793	.154	2.21***	1.64	2.99		Dedication	1.391	.210	4.02***	2.66	6.07	
Absorption	−.235	.133	.79	.61	1.03	.272	Absorption	.262	.114	1.30*	1.04	1.62	.457
Intention to leave hospital: yes (1) versus no (0)							Intention to leave profession: yes (1) versus no (0)						
	B	SE	OR	95% C.I.		adjR^2^		B	SE	OR	95% C.I.		adjR^2^
*N* = 1083				Lower	Upper		*N* = 1085				Lower	Upper	
Generations							Generations			***			
Generations (Z)	.562	.827	1.75	.35	8.88		Generations (Z)	1.461	.802	4.31	.90	20.78	
Generations (Y)	1.04	.411	2.83*	1.27	6.33		Generations (Y)	2.611	.556	13.60***	4.58	40.45	
Generations (X)	.506	.432	1.66	.71	3.87		Generations (X)	1.768	.568	5.86**	1.92	17.83	
Gender male)	.416	.302	1.52	.84	2.74	.053	Gender (male)	−.186	.327	.83	.44	1.58	.085
Professional Category			*				Professional Category			**			
Category (1)	−.914	.310	0.40**	.22	.74		Category (1)	1.529	.471	4.61**	1.83	11.60	
Category 2)	.-788	.470	0.46	.18	1.14	.068	Category 2)	1.171	.569	3.23*	1.06	9.83	.112
Social capital	−.901	.237	0.41***	.26	.65		Social capital	−.234	.231	.79	.50	1.24	
Decision latitude	−.088	.442	0.92	.39	2.18		Decision latitude	−.148	.407	.86	.39	1.92	
Workload	.213	.280	1.24	.72	2.14	.194	Workload	.157	.258	1.17	.71	1.94	.168
Vigor	−.018	.152	0.98	.73	1.32		Vigor	.105	.139	1.11	.85	1.46	
Dedication	−.647	.176	0.52***	.37	.74		Dedication	−.617	.163	.54***	.39	0.74	
Absorption	.039	.157	1.04	.76	1.42	.265	Absorption	−.20	.137	.82	.63	1.07	.260

***P-value < .001; **P-value < .01; *P-value < .05; OR = Odds Ratio 95% CI [lower and upper bound]; Adjusted R2 reported additionally; Baby boomers as indicator; Female as indicator; Medical staff as indicator, nursing staff/category 1, healthcare staff/category 2; Social capital, decision latitude, workload, and work engagement dimensions mean value

**Table 3:** Hierarchical regression analyses with personal characteristics (1), category (2), social capital, decision latitude and workload (3) and work engagement dimensions (4) (explanatory variables) and quality of care unit and hospital (dependent variables)

Quality of care unit: good or excellent (1) versus fair poor (0)							Quality of care unit: excellent (1) versus good or fair or poor (0)						
	B	SE	OR	95% C.I.		adjR^2^		B	SE	OR	95% C.I.		adjR^2^
*N* = 1048				Lower	Upper		*N* = 1070				Lower	Upper	
Generations			**				Generations			***			
Generations (Z)	−.862	.471	.42	.17	1.06		Generations (Z)	−1.782	.451	.17***	.07	.41	
Generations (Y)	−1.048	.300	.35***	.20	.63		Generations (Y)	−1.037	.219	.36***	.23	.54	
Generations (X)	−.417	.316	.66	.36	1.23		Generations (X)	−.223	.198	.80	.54	1.18	
Gender (male)	−.276	.243	.76	.47	1.22	.047	Gender (male)	−.108	.200	.90	.61	1.33	.070
Professional Category			*				Professional Category			***			
Category (1)	−.574	.297	.56	.32	1.01		Category (1)	−1.122	.207	.33***	.22	.49	
Category 2)	−.985	.373	.37**	.18	.78	.056	Category 2)	−1.401	.320	.25***	.13	.46	.118
Social capital	1.518	.208	4.56***	3.04	6.86		Social capital	1.532	.177	4.63***	3.27	6.55	
Decision latitude	.155	.330	1.17	.61	2.23		Decision latitude	.676	.256	1.97***	1.19	3.25	
Workload	−.408	.212	.67	.44	1.01	.245	Workload	−.307	.163	.74	.53	1.01	.290
Vigor	.303	.109	1.35**	1.09	1.68		Vigor	−.114	.109	.89	.72	1.11	
Dedication	.149	.138	1.16	.89	1.52		Dedication	.232	.145	1.26	.95	1.68	
Absorption	−.057	.116	.95	.75	1.19	.276	Absorption	.046	.099	1.05	0.86	1.27	.295
Quality of care hospital: improved or certainly improved (1) versus deteriorated or certainly deteriorated (0)							Quality of care hospital: certainly improved (1) versus improved or deteriorated or certainly deteriorated (0)						
	B	SE	OR	95% C.I.		adjR^2^		B	SE	OR	95% C.I.		adjR^2^
*N* = 1056				Lower	Upper		*N* = 1054				Lower	Upper	
Generations							Generations						
Generations (Z)	1.048	.446	2.85*	1.19	6.84		Generations (Z)	−.611	.542	.54	.19	1.57	
Generations (Y)	−.119	.216	.89	.58	1.36		Generations (Y)	−.310	.307	.73	.40	1.34	
Generations (X)	−.131	.214	.88	.58	1.33		Generations (X)	−.198	.287	.82	.47	1.44	
Gender (male)	.264	.209	1.30	.86	1.96	.024	Gender (male)	.002	.278	1.00	.58	1.73	.008
Professional Category			***				Professional Category						
Category (1)	−1.276	.269	.28***	.17	.47		Category (1)	−.0660	.297	.94	.52	1.68	
Category 2)	−.741	.361	.48*	.24	;97	.070	Category 2)	−.258	.463	.77	.31	1.91	.010
Social capital	.679	.162	1.97***	1.44	2.71		Social capital	.815	.263	2.26**	1.42	3.59	
Decision latitude	0,426	.264	1.53	.91	2.57		Decision latitude	1.349	.343	3.86***	1.97	7.55	
Workload	− 1059	.173	.35***	.25	0.49	.191	Workload	−.459	.226	.63*	.41	.98	.129
Vigor	0.139	.094	1.15	.96	1.38		Vigor	.316	.199	1.37	.93	2.03	
Dedication	−.038	.118	.96	.76	1.21		Dedication	−.147	.247	.86	.53	1.40	
Absorption	.104	.094	1.11	.92	1.33	.204	Absorption	.327	.162	1.39*	1.01	1.91	.166

***P-value < .001; **P-value < .01; *P-value < .05; OR = Odds Ratio 95% CI [lower and upper bound]; Adjusted R2 reported additionally; Baby boomers as indicator; Female as indicator; Medical staff as indicator, nursing staff/category 1, healthcare staff/category 2; Social capital, decision latitude, workload and work engagement dimensions mean value

**Table 4:** Hierarchical regression analyses with personal characteristics (1), category (2), social capital, decision latitude and workload (3) and burnout dimensions (4) (explanatory variables) and job satisfaction; intention to leave hospital and profession (dependent variables)

Job satisfaction: satisfied or very satisfied (1) versus dissatisfied or very dissatisfied (0)							Job satisfaction: very satisfied (1) versus satisfied or dissatisfied or very dissatisfied (0)						
	B	SE	OR	95% C.I.		adjR^2^		B	SE	OR	95% C.I.		adjR^2^
*N* = 1039				Lower	Upper		*N* = 1039				Lower	Upper	
Generations							Generations						
Generations (Z)	.235	.607	1.27	.39	4.16		Generations (Z)	.379	.346	1.46	.74	2.87	
Generations (Y)	−.555	.317	.57	.31	1.07		Generations (Y)	.066	.223	1.07	.69	1.66	
Generations (X)	−.180	.326	.84	.44	1.58		Generations (X)	−.169	.220	.84	.55	1.30	
Gender (male)	−.192	.279	.83	.48	1.43	.021	Gender (male)	.020	.205	1.02	.68	1.52	.021
Professional Category			**				Professional Category						
Category (1)	−.932	.344	.39**	.20	.77		Category (1)	−.341	.232	.71	.45	1.12	
Category 2)	−.022	.531	.98	.35	2.77	.005	Category 2)	−.094	.325	.91	.48	1.72	.013
Social capital	1.034	.216	2.81***	1.84	4.30		Social capital	.934	.174	2.54***	1.81	3,57	
Decision latitude	.704	.316	2.02	1.00	4.10		Decision latitude	1.989	.276	7.31***	4.26	12.56	
Workload	−.190	.272	0.83	.49	1.41	.191	Workload	−.504	.196	.60*	.41	.89	.286
Emotional exhaustion	−.681	.125	.51***	.40	.65		Emotional exhaustion	−.816	.116	.44***	.35	.56	
Depersonalisation	.192	.142	1.21	.92	1.60		Depersonalisation	.084	.142	1,09	.82	1.44	
Personal accomplishment	−.028	.150	.97	.73	1,.30	.251	Personal accomplshment	.533	.137	1.71***	1.30	2.23	.387
Intention to leave hospital: yes (1) versus no (0)							Intention to leave profession: yes (1) versus no (0)						
	B	SE	OR	95% C.I.		adjR^2^		B	SE	OR	95% C.I.		adjR^2^
*N* = 1039				Lower	Upper		*N* = 1042				Lower	Upper	
Generations							Generations			***			
Generations (Z)	.181	.835	1.20	.23	6.16		Generations (Z)	1.023	.799	2.78	.58	13.33	
Generations (Y)	.781	.428	2.18	.94	5.06		Generations (Y)	2.175	.548	8.81***	3,.01	25.77	
Generations (X)	.487	.441	1.63	.69	3.86		Generations (X)	1.427	.562	4.17*	1.38	12.54	
Gender (male)	.649	.306	1.91*	1.05	3.49	.052	Gender (male)	.160	.330	1,.17	.61	2.24	.078
Professional Category							Professional Category			***			
Category (1)	−.382	.324	.68	.36	1.29		Category (1)	2.280	.568	9.78***	3.21	29.78	
Category 2)	−.286	.503	.75	.28	2.02	.062	Category 2)	2.023	.665	7.56**	2.06	27;85	.112
Social capital	−1.055	.243	.35***	.22	.56		Social capital	−.394	.238	.67	;42	1.08	
Decision latitude	−.185	.440	.83	.35	1.97		Decision latitude	−.685	.413	.50	.22	1.13	
Workload	−.259	.334	.77	.40	1.49	.184	Workload	−.419	.314	.66	.36	1.22	.183
Emotional exhaustion	.544	.152	1.72***	1.28	2.32		Emotional exhaustion	.667	.147	1.95***	1.46	2.60	
Depersonalisation	.117	.161	1.12	.82	1.54		Depersonalisation	.041	.159	1.04	.76	1.42	
Personal accomplishment	−.124	.171	.88	.63	1.24	.242	Personal accomplishment	−.134	.170	.88	.63	1.22	.254

***P-value < .001; **P-value < .01; *P-value < .05; OR = Odds Ratio 95% CI [lower and upper bound]; Adjusted R2 reported additionally; Baby boomers as indicator; Female as indicator; Medical staff as indicator, nursing staff/category 1, healthcare staff/category 2; Social capital, decision latitude, workload and burnout dimensions mean value

In the hierarchical regression models with the work engagement and burnout dimensions we identified several significant associations with studied variables and outcomes. In particular, social capital and decision latitude were positive and workload (block - 3) was negative associated with staff that was *very* satisfied with explained variances in block 3 of 27,5% and 28,6% (see Tables 2, 3 and 4). Moreover, quality of care at the unit assessed as *excellent* was positive associated with social capital and decision latitude but not with workload with explained variances in block 3 of 29% and 27,5% (see Tables 3, 4 and 5). Regression models with engagement dimensions showed positive associations of social capital and decision latitude and negative associations of workload with *certainly* improved quality of care in the hospital with explained variances in block 3 of 12.9% (see Table 3). In addition, intention to leave the hospital and the profession were negative associated with dedication (block – 4) with total explained variances for both variables of 26%. (see Tables 2, 3 and 4). Instead, in the hierarchical regression models with the burnout dimensions emotional exhaustion (block – 4) was positive associated with intention to leave the hospital and intention to leave the profession with total explained variances were 24.2% and 25.4%, respectively (see Tables 3, 4 and 5).

**Table 5:** Hierarchical regression analyses with personal characteristics (1), category (2), social capital, decision latitude and workload (3) and burnout dimensions (4) (explanatory variables) and quality of care unit and hospital (dependent variables)

Quality of care unit: good or excellent (1) versus fair poor (0)							Quality of care unit: excellent (1) versus good or fair or poor (0)						
	B	SE	OR	95% C.I.		adjR^2^		B	SE	OR	95% C.I.		adjR^2^
*N* = 1032				Lower	Upper		*N* = 1032				Lower	Upper	
Generations			**				Generations			***			
Generations (Z)	−.663	.491	.52	.20	1.35		Generations (Z)	−1.749	.452	.17***	.07	.42	
Generations (Y)	−.990	.312	.37**	.20	.69		Generations (Y)	−.890	.221	.41***	.27	.63	
Generations (X)	−.418	.326	.66	.35	1.25		Generations (X)	−.194	.199	.82	.56	1.22	
Gender (male)	−.226	.252	.80	.49	1.31	.050	Gender (male)	−.134	.203	.87	.59	1.30	.072
Category			*				Category			***			
Category (1)	−.687	.306	.50*	.28	.92		Category (1)	−1.249	.219	.28***	.19	.44	
Category 2)	−1.057	.392	.35**	.16	.75	.056	Category 2)	−1.653	.343	.19***	.10	.38	.120
Social capital	1.424	.209	4.15***	2.76	6.26		Social capital	1.329	.178	3.78***	2.66	5.35	
Decision latitude	.156	.331	1.17	.61	2.24		Decision latitude	.659	.259	1.93*	1.16	3.21	
Workload	−.041	.252	.96	.59	1.57	.225	Workload	−.130	.187	.88	.61	1.27	.275
Emotional exhaustion	−.337	.115	.71**	.57	.90		Emotional exhaustion	−.038	.097	.96	.80	1.17	
Depersonalisation	−.126	.131	.88	.68	1.14		Depersonalisation	−.442	.142	.64**	.49	.85	
Personal accomplishment	.188	.188	1.21	.94	1.56	.259	Personal accomplishment	.051	.117	1.05	.84	1.32	.293
Quality of care hospital: improved or certainly improved (1) versus deteriorated or certainly deteriorated (0)							Quality of care hospital: certainly improved (1) versus improved or deteriorated or certainly deteriorated (0)						
	B	SE	OR	95% C.I.		adjR^2^		B	SE	OR	95% C.I.		adjR^2^
*N* = 1021				Lower	Upper		*N* = 1021				Lower	Upper	
Generations			*				Generations						
Generations (Z)	1.259	.474	.17***	.07	.42		Generations (Z)	−.502	.539	.61	.21	1.74	
Generations (Y)	−.094	.221	.41***	.27	.63		Generations (Y)	−.261	.320	.77	.41	1.44	
Generations (X)	−.087	.219	.82	.56	1.22		Generations (X)	−.149	.291	.86	.49	1.53	
Gender (male)	0.334	.215	.87	.59	1.30	.072	Gender (male)	.116	.284	1.12	.65	1.96	.011
Professional Category			***				Professional Category						
Category (1)	−1.394	.284	.28***	.19	.44		Category (1)	−.121	.306	.89	.49	1.61	
Category 2)	−1.123	.372	.19***	.10	.38	.120	Category 2)	−1.042	.586	.35	.11	1.11	.021
Social capital	.601	.166	3.78***	2.66	5.35		Social capital	.443	.240	1.56	.97	2.49	
Decision latitude	.500	.264	1.93*	1.16	3.21		Decision latitude	1.414	.360	4.11***	2.03	8.33	
Workload	−.860	.198	.88	.61	1.27	.275	Workload	−.325	.259	.72	.44	1.20	.115
Emotional exhaustion	−.142	.091	.96	.80	1.17		Emotional exhaustion	.005	.147	1.01	.75	1.34	
Depersonalisation	−.283	.114	.64**	.49	.85		Depersonalisation	−.552	.231	.58*	.37	.91	
Personal accomplishment	.023	.110	1.05	.84	1.32	.293	Personal accomplishment	.354	.203	1.42	.96	2.12	.144

***P-value < .001; **P-value < .01; *P-value < .05; OR = Odds Ratio 95% CI [lower and upper bound]; Adjusted R2 reported additionally; Baby boomers as indicator; Female as indicator; Medical staff as indicator, nursing staff/category 1, healthcare staff/category 2; Social capital, decision latitude, workload and burnout dimensions mean value

## Discussion

The study confirms and extends previous findings on nurse populations showing positive impact on staff’ job outcomes and assessed quality of care by *balanced work characteristics* such as favorable rated social capital, decision latitude and workload in healthcare staff and medical staff as well [14, 25, 41]. In the hierarchical regression analyses we identified significant favorable impact of all three work characteristics on respondents who rated their job as very satisfied and the quality at the unit as excellent in both engagement and burnout models. In contrast with the burnout model, certainly improved quality of the hospital was associated with the three work characteristics in the engagement model. We suggest that the workplace conditions were more balanced and that these respondents could be seen as *ambassadors* of the study hospital. Instead, it seems that in the other models the workplace conditions were not fully balanced. Qualitative research design could reveal the differences in workplace conditions among nursing staff, healthcare staff and medical staff for each study outcome.

Intention to leave the hospital and the profession were positive associated with emotional exhaustion and negative associated with dedication in burnout and engagement models, respectively. Previous confirmed structural equation models showed social capital and decision latitude as predictors of emotional exhaustion and dedication, respectively [23]. It seems that these healthcare workers in comparison with their colleagues lost their energy and involvement, previously described as cycles of loss versus the cycles of gains. The first through strain processes, the latter through motivational processes [16–18]. Moreover, although not measured leadership at hospital level as well as unit level along with mutual values and goals between these leadership levels are key for favorable work characteristics [14].

Aged studied in generational groups (X and Y) and professional category (nursing staff and healthcare staff) were associated with turnover intentions (mainly the profession) and less favorable assessed quality of care, respectively. These results are in line with previously reported studies demonstrating that intergenerational differences affect occupational well-being, performance, productivity and patient safety [52], and should be considered to enhance and support psychosocial work conditions (5).

Total explained variances of studied models ranged from 14.4 to 45.7%.

In the study hospital the main focus was to implement a cultural change in leadership style and interdisciplinary collaboration. Meting standards through accreditation came later through government obligation and created some confusion. JCI-accreditation requirements, although accepted by nursing staff in the study hospital as of added value because of standardization of processes, were perceived as more top down implementation as compared to PW program [43]. A systematic review did not find evidence to support accreditation of hospitals being linked to measurable changes in quality of care. The authors referred that due to heterogeneity of study design and methods much uncertainly remains regarding its putative effect. Furthermore, accreditation programs require substantial financial and labor investments because of the distraction on healthcare teams from their primary clinical goals [12]. Therefore, authors recommend more research on the clinical impact as well as to weigh the transactional opportunity and financial costs of accreditation against other financial investments in quality improvement interventions.

Unlike other professionals, physicians are primarily educated to be clinicians rather than a leader and team member and mainly focused on their clinical work then the need to work with co-workers and patients who have different visions of how the organization of the hospital should operate. Therefore, because of the disconnection between their training and expectations and the reality, as well as fewer resources and tighter budgets, physicians are prone for conflicts, personal and professional strain or higher levels of burnout as shown in our findings [54]. In spite of the success of the Inter-professional Collaboration in Healthcare (IPCIHC) modules provided in the undergraduate programs at the University of Antwerp (Belgium), there are still great challenge ahead in educating future healthcare providers to enact positive behaviors in inter-professional collaboration [55].

This cross-sectional study within a longitudinal study design, guiding quality improvements strategies, must be interpreted with caution and related to the studied academic centre context as well. Moreover, we recommend in future studies to use multilevel analyses investigating the impact at interdisciplinary team level previous shown as relevant in nursing teams [41, 56]. Lower response rates of physician study samples as shown in our study are well known and investigated such as the potential impact of incentives [57], recommended efforts to increase overall response among this hard-to-reach population [58] as well as methods in general used to boost online survey response rates [59]. Researchers debate and study the importance of response bias such as survey methodologists who have found that low response rates do not necessarily bias results [60]. As aforementioned argued qualitative research design could support and extended our study finding in-depth.

### Relevance and implication for practices

Implementation sciences identified necessary key constructs in organizational inner settings such as culture, leadership engagement, available resources, and access to information and knowledge [61]. Moreover, the Systems Engineering Initiative for Patient Safety or SEIPS-model describes adaptation as feedback mechanism that explains how dynamic systems evolve in planned and unplanned ways [62]. These mechanisms that support continuous improvement efforts structurally need to be aligned between hospital and team governance level. High reliability, a paradigm in the patient safety movement that focused on commitment and anticipation, is rather based on deference to *expertise* instead of authority and *customer-focused* instead of physician-focused. Therefore, physicians in collaboration interdisciplinary should take the lead in quality improvements instead holding organization responsible [63]. Physicians of the study hospital intent to support quality and patient safety improvements including JCI-standards declared in a hospital policy statement (2017). Moreover, long-term strategies supported by a Magnet Recognition® journey could guide hospital governance and policy as well as clinical teams achieving a culture of learning, adaptation and resilience [14, 64]. Healthcare organizations are challenged maximizing their capacities and abilities to solve and find answers for continuous changing needs of patients and their families. Therefore, hospitals in their effort to achieve attractive and productive workplaces for nursing staff, healthcare staff and medical staff should monitor and evaluate, for each professional group within interdisciplinary collaborations, balanced work characteristics in order to achieve and sustain state-of-the-art outcomes. Nevertheless, professionals bear responsibility and involvement, each in their capacities and their specific roles, in case of concerned and unbalanced work characteristics.

## Conclusion

Our study confirms the relevance of *balanced work characteristics* on nursing staff, healthcare staff and medical staff’ job outcomes and perceived quality of care in an academic setting focused on quality improvements structurally. Engaging and committing staff to promote excellent patient outcomes in daily interdisciplinary practice works when clear frameworks, methods and resources are supported by hospital governance and policy structure that makes outcomes visible and accountable.
